# To pretrain or not? A systematic analysis of the benefits of pretraining in diabetic retinopathy

**DOI:** 10.1371/journal.pone.0274291

**Published:** 2022-10-18

**Authors:** Vignesh Srinivasan, Nils Strodthoff, Jackie Ma, Alexander Binder, Klaus-Robert Müller, Wojciech Samek

**Affiliations:** 1 Department of Artificial Intelligence, Fraunhofer Heinrich Hertz Institute, Berlin, Germany; 2 School of Medicine and Health Services, Oldenburg University, Oldenburg, Germany; 3 BIFOLD - Berlin Institute for the Foundations of Learning and Data, Berlin, Germany; 4 Singapore Institute of Technology, ICT Cluster, Singapore, Singapore; 5 Department of Informatics, Oslo University, Oslo, Norway; 6 Department of Electrical Engineering and Computer Science, Technische Universität Berlin, Berlin, Germany; 7 Department of Artificial Intelligence, Korea University, Seoul, South Korea; 8 Max Planck Institute for Informatics, Saarbrücken, Germany; Universita degli Studi di Perugia, ITALY

## Abstract

There is an increasing number of medical use cases where classification algorithms based on deep neural networks reach performance levels that are competitive with human medical experts. To alleviate the challenges of small dataset sizes, these systems often rely on pretraining. In this work, we aim to assess the broader implications of these approaches in order to better understand what type of pretraining works reliably (with respect to performance, robustness, learned representation etc.) in practice and what type of pretraining dataset is best suited to achieve good performance in small target dataset size scenarios. Considering diabetic retinopathy grading as an exemplary use case, we compare the impact of different training procedures including recently established self-supervised pretraining methods based on contrastive learning. To this end, we investigate different aspects such as quantitative performance, statistics of the learned feature representations, interpretability and robustness to image distortions. Our results indicate that models initialized from ImageNet pretraining report a significant increase in performance, generalization and robustness to image distortions. In particular, self-supervised models show further benefits to supervised models. Self-supervised models with initialization from ImageNet pretraining not only report higher performance, they also reduce overfitting to large lesions along with improvements in taking into account minute lesions indicative of the progression of the disease. Understanding the effects of pretraining in a broader sense that goes beyond simple performance comparisons is of crucial importance for the broader medical imaging community beyond the use case considered in this work.

## Introduction

The role of computer vision algorithms based on deep learning in medical imaging in the form of decision support systems has increased steadily in the past few years [1–7]. There is an enormous amount of data that is being produced on a daily basis from different areas using different imaging modalities such as MRI, CT, microscopy, etc., leading to an unprecedented potential for machine learning algorithms. However, while there exists a lot of data, it is usually not prepared to be used for research in machine learning. In particular, it is often unlabeled as the labeling process is expensive and time-consuming or sometimes medical experts may not agree on the appropriate label.

A practitioner using Deep Neural Networks (DNN) for the task of medical imaging, is faced with a plethora of options when it comes to the training methodology for the DNN. Several factors can influence the decision making process including, but not limited to the size, noise level and quality of the dataset at hand, computational resources available and robustness of the trained DNN. Transfer learning, i.e. pretraining models on a large corpora of natural images has been found to be beneficial for improvements in performance along with speeding up convergence on downstream tasks such as medical imaging [1, 8]. A straightforward way of utilizing transfer learning is to finetune a model that has been initially trained on ImageNet [9] on the medical dataset.

Other common state-of-the-art methods in machine learning are *supervised-learning* methods, i.e. models that are trained with labeled data, opposed to other methods that require only some or even no labeled data such as *semi-supervised* or *self-supervised learning*. Fortunately, the field of self-supervised learning has recently advanced significantly [12–15], which gives rise to hope for a successful deployment of machine learning in medical applications without relying on overly large amounts of labeled data. A first result in this regard was obtained in [6, 16, 17] where the authors showed that pretraining using self-supervision helps to improve the models for chest x-ray classification [18], dermatology condition classification [19] and COVID-19 deterioration prediction [17].

With widespread adoption of transfer learning in medical imaging, it becomes essential to explore the differentiating features of the various training methodologies—supervised or self-supervised. [1] observe the effects of pretraining on the speed of convergence and feature representations learned, but only in a supervised learning setting. [8] find that pretrained models from ImageNet provide improvements in quality of the features learned performance as well as improvements in performance on diverse downstream datasets. Despite the benefits of transfer learning, it has however remained unclear what transfer learning, especially with self-supervised learning actually exploits when making a prediction. For this (as we will see) simply looking at performance metrics like classification accuracy or area under the operating curve (AUC) is not sufficient. The potential advantages of using self-supervised methods over supervised methods for medical imaging beyond such performance metrics thus remain a challenging object of study.

In this contribution, we demonstrate for diabetic retinopathy (DR) as a particular medical imaging use case, that going beyond metrics of predictive performance is mandatory. We further analyze robustness to statistical variations of the data. Furthermore we validate previous results on smaller data sets which are of ubiquitous interest to practitioners in medical data science.

To this end, we perform a detailed study of what is being learned by the different training methodologies available to train a DNN for medical imaging. Broadly, the training methodologies will be categorized into two types:

- Fully supervised (FS)
- Self-supervised with contrastive learning (CL)

along with two types of initialization of the weights before training on the medical dataset:

- Initialization with no external data (IWNE)
- Initialization from ImageNet (IFI)

**Fig 1:**
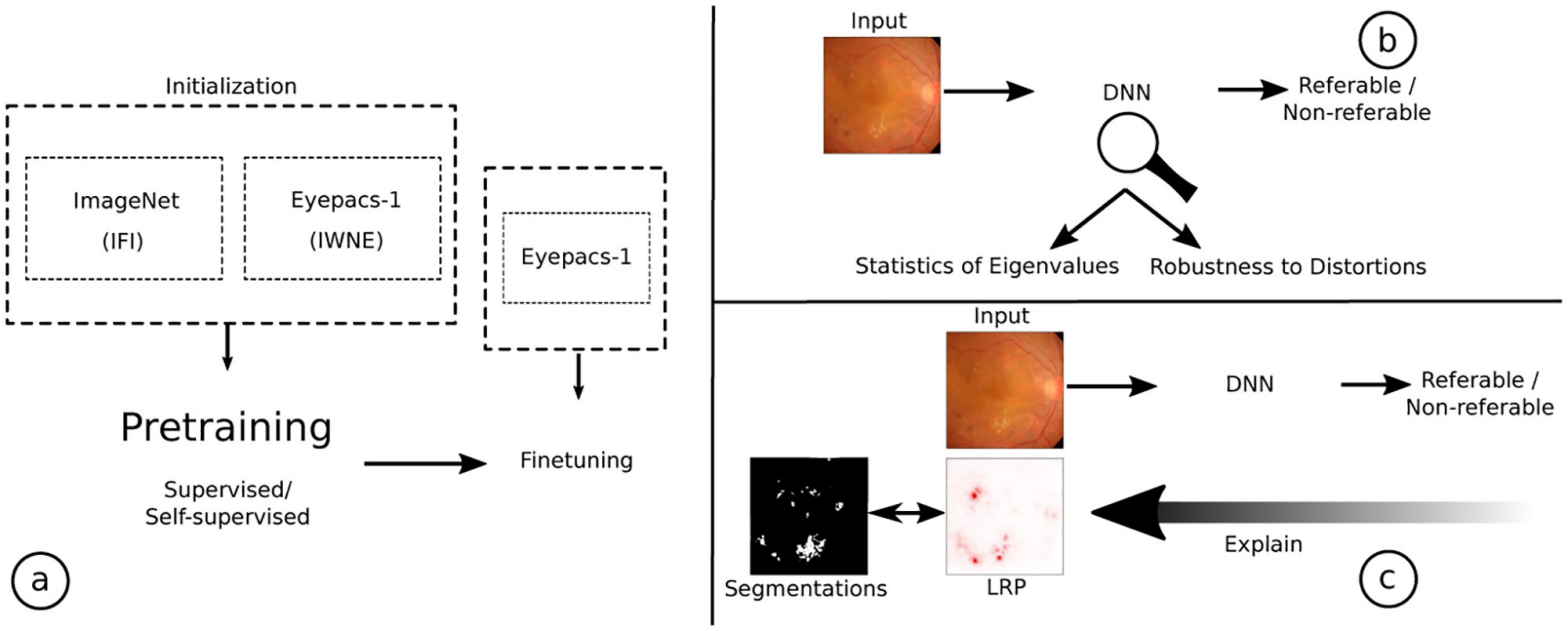
Overview of the experiments presented in this work. a) shows the different pretraining strategies: Initialization from ImageNet (IFI) [9] and Initialization without any external data (IWNE), i.e. pretraining only on Eyepacs-1 datasets [10]. Such a pretraining step can be performed either in a supervised or a self-supervised manner. This is followed by finetuning on the Eyepacs-1 dataset. b) investigates the statistics of the eigenvalues of the feature representations learned by the different methods which lead to increased robustness to distortions. c) shows the experiments we perform using the Indian Diabetic Retinopathy Image Dataset (IDRiD) challenge data [11] to quantitatively evaluate the cues learned.

The focus of this paper is to study the effects of training the DNN using these strategies and evaluate the benefits. Fig 1 gives an overview of our contributions which are as follows:

1. We evaluate the performance of the four different training strategies: supervised and self-supervised models using models trained with or without using external data for pretraining in detecting diabetic retinopathy in retinal images. We find that IFI helps in achieving significant gain in performance, especially when a limited amount of the downstream (medical) labeled dataset is used. IFI-CL provides a further increase in performance.
2. Given that IFI is beneficial in terms of performance, we investigate what makes them better by analyzing the eigenvalue spread of the activations on the hidden layers. We find that the redefined conditioning number for the IFI models is lower than that of IWNE models for the initial layers that are important for learning diverse and effective feature representations from the input. IFI makes the eigenvalue spread of the activations of the first hidden layer broader, implying that a wider range of kernels fire for a given input. In both IWNE as well as IFI models, we show that CL achieves broader eigenvalue spread compared to its supervised counterparts.
3. Using explainability of DNNs, we investigate what the different models look at in the input for making a decision. With the help of ground-truth segmentation maps available for diabetic retinopathy on the IDRiD challenge [11], we study in a quantitative manner what information was used by the models to make the prediction. We find that IWNE-FS overfits to large lesions like hard exudates and ignores smaller lesions to predict the disease. IFI models show significantly reduced tendency to overfit to one particular type of lesions. Especially IFI-CL is able to consider a wider range of lesions to make an accurate prediction for the disease.

## Related work

### Diabetic retinopathy

DNNs have seen wide adoption for the task of DR assessment in [2, 3, 20–42] among others. While some methods train their model from scratch [20, 21, 32, 35, 43], IFI models have predominantly achieved higher performance [2, 3, 23, 26, 30, 40]. Some methods also perform their training on large private data [2, 20, 24, 29, 33]. A reproduction study of [2] was performed by [3] showing difficulty in achieving similar performance for DR when trained on publicly available datasets. Systematic study of using uncertainty measures for DR were also conducted by [43, 44]. While [22] studied the probability maps with ground-truth segmentation maps to ascertain what the DNN prediction was looking for [45], studied a computer-assisted setting with explanation methods for deep learning models in grading for DR. There is, however, no dedicated study on the implications of different training methodologies.

### Supervised vs. self-supervised learning

Self-supervised learning has been utilized in a wide range of biomedical applications including chest x-rays [4–6, 17], diabetic retinopathy [47, 48], COVID-19 detection [17] etc. In spite of the improvements shown by self-supervised learning [49], find that self-supervised models behave quite similarly to their supervised counterparts in many aspects of robustness. Self-supervised models report a slightly higher performance gain over their supervised counterparts on medical imaging [4, 6]. Recent works show the generalizing capabilities of self-supervised learning on chest x-rays [50]. The improvements and benefits still need to be rigorously investigated to ascertain the limits of using self-supervised learning on real-life healthcare applications.

### IWNE vs IFI

Pretraining on ImageNet dataset (i.e. IFI), either supervised or self-supervised, is considered an effective strategy [4–6, 8, 51–56]. Several benefits have been attributed to pretraining including robustness [8, 51–54], generalization [57, 58], finding sparser subnetworks from the original [59] and also speed up in convergence on the downstream task [1, 8]. Using IFI for DR has been widely adopted owing to benefits in performance [1–3, 23, 26, 32, 60]. The performance benefits of pretraining have been observed even on diverse datasets which seem distant from the ImageNet dataset [8]. The benefits of pretraining can be attributed to effective feature extracting capability of pretrained models in the lower layers [1, 8]. Although, it is unclear how this translates to a DNN being used for a downstream task after finetuning. While the above mentioned methods investigate supervised learning, we make a comparative study of IWNE vs IFI along with FS vs CL and their combinations to understand their differentiating features.

## Materials & methods

### Datasets

We focus on diabetic retinopathy (DR) as a use case for our investigations and solely work on publicly available datasets, which are summarized in Table 1.

**Table 1:** Diabetic retinopathy datasets used for this study.

Dataset	# instances	# patients
EyePacs-1 (EyP) [10]	88,702	44,351
Messidor-2 [46]	1,744	872
IDRiD [11]	80	-

We make use of the Eyepacs-1 dataset [10], which is available from a former Kaggle challenge. The images are graded from a scale of 0 to 4 (0: no DR, 1: mild DR, 2: moderate DR, 3: severe DR, 4: proliferative DR) according to the International Clinical Diabetic Retinopathy (ICDR) severity scale. DR advances from a healthy eye to a proliferate one slowly and may also take years. However, this transition is discrete and often goes undetected to worsen into a proliferate DR. Hence, it is essential that this progression is detected and a timely medical diagnosis is performed. In our experiments, we train the models to perform the quinary classification using all the five grades. During inference, we formulate the outputs predicted by the model to a binary classification by summing up the output neurons corresponding the the two labels, i.e. *healthy* classes [0–2] and *disease* classes [3–4]. Following the summation, we apply softmax activation to map the outputs to the range of [0, 1] to obtain output probabilities. This binary class formulation is consistent with referable DR (rDR) classification in [2, 3].

The Eyepacs-1 dataset [10] consists of 35216 images in the training set and 53576 in the test set. We utilize non-overlapping set of around 15% of the training set as the validation set. We train all our different methods on the training set of Eyepacs-1 dataset and evaluate the performance of the models on two datasets—test set of Eyepacs-1 and Messidor-2 [46]. Messidor-2 dataset [46] is a benchmark dataset consisting of 1744 images that are 100% gradable. The evaluation on the Messidor-2 dataset is supposed to measure the generalization performance of the algorithms since the dataset is not used for training and was collected under different conditions, at a different geographical location and with different hardware. Hence, we use all the images of this dataset for testing. We report the AUC for the binary rDR classification task on the respective test sets of each dataset.

### Models & training procedures

We compare the four training setups which are eventually trained on the DR target dataset.

- *Initialization With No External Data (IWNE)*
  - **FS**: supervised training on the DR dataset starting from randomly initialized weights.
  - **CL**: self-supervised pretraining on the target domain and finetuning also on the same dataset using labeled data.
- *Initialization From ImageNet Data (IFI)*
  - **FS**: supervised training on the DR dataset starting from supervised ImageNet-pretrained weights.
  - **CL**: self-supervised pretraining on ImageNet dataset and finetuning on the DR dataset using labeled data.

For comparability, we fix the architecture and use a Resnet50 [61] model for all our experiments. In the self-supervised setting, we pretrain the models using MoCoV2 strategy [62]. For the supervised pretraining, we use the ImageNet-pretrained model provided by torchvision. The IWNE models are trained for 500 epochs with a learning rate of 10^−4^. Pretrained models have shown to be faster at convergence than the models trained from scratch [1, 8]. Hence, we finetune the IFI models starting from ImageNet-pretrained weights for 50 epochs with a learning rate of 10^−3^. The IFI models use the same mean and standard deviation of the ImageNet dataset while IWNE models use mean and standard deviation computed from the training set of the Eyepacs-1 dataset. The AdamW optimizer [63] with weight decay was used in all the settings. The best models in each training run was chosen based on the maximum AUC score achieved on the validation set and this model was used for inference on the test.

## Experiments & results

### Quantitative performance

**Fig 2:**
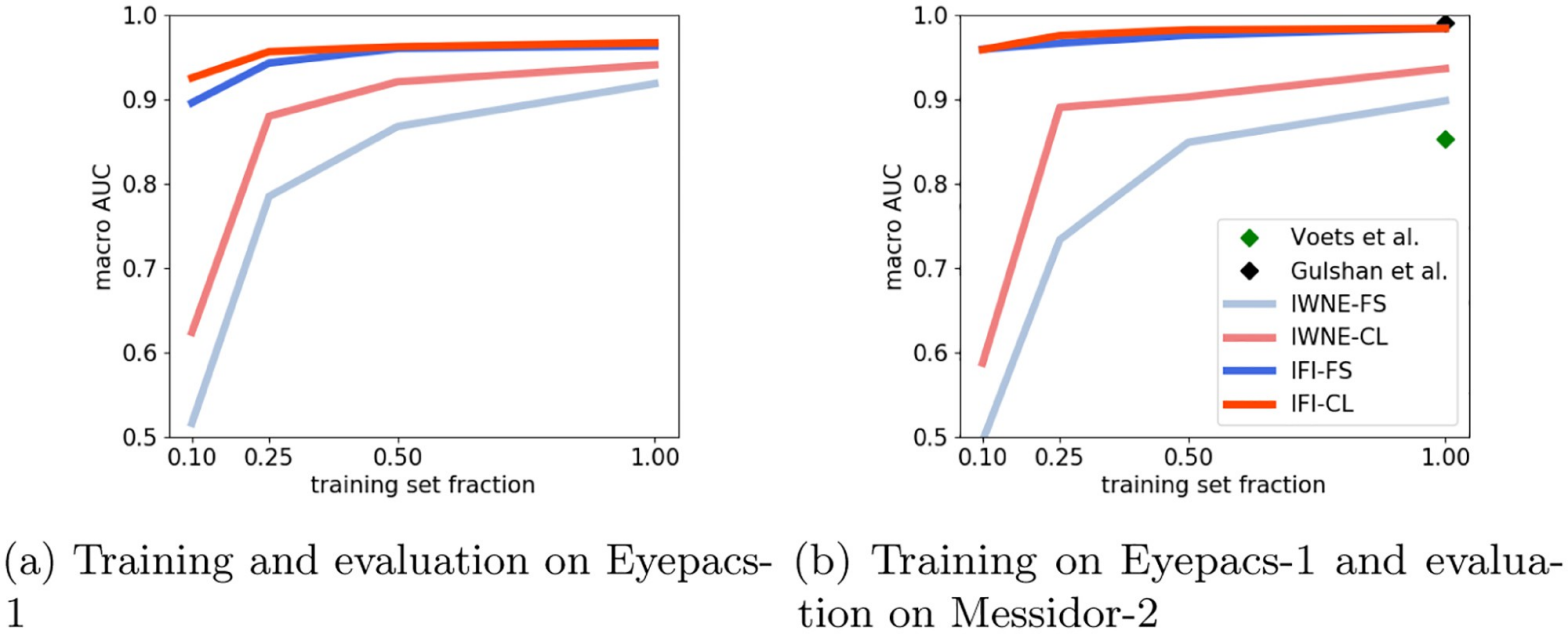
Classification performance on Eyepacs-1 and Messidor-2 dataset for referable DR. The x-axis in both the subfigures correspond to the fraction of the labeled training set from the Eyepacs-1 dataset used for the downstream training of the four different training procedures. The state-of-the-art method for DR—Voets et al. [3] and Gulshan et al. [2] are shown as green and black diamonds for training with the full dataset for the Messidor-2 dataset. (a) Training and evaluation on Eyepacs-1. (b) Training on Eyepacs-1 and evaluation on Messidor-2.

We evaluate the performance of the different methods discussed in Section Models & Training Procedures in terms of AUC. Each model was trained on the full dataset and on various fractions of the training set down to a fraction of 10% labeled samples. Fig 2 shows the final AUC of the binary classification for rDR. We find largely consistent results in terms of the ranking and overall behavior of the different training procedures between evaluation on a subset of the Eyepacs-1 dataset used for training and an evaluation on the external Messidor-2 dataset, which is a reassuring sign that our results generalize across datasets. The best-performing method across all the training set fractions is IFI-CL, i.e. finetuning a model that was trained in a self-supervised fashion on ImageNet data, closely followed by IFI-FS, corresponding to the standard training methodology in medical imaging, where a model pretrained on ImageNet is finetuned on the target dataset. The results for the IWNE-CL model, i.e. self-supervised pretraining in target (DR) domain are weaker than the former two results. This trend is again followed at lower training set fractions where the model is trained with reduced fractions of the labeled dataset. A training set fractions of 1.0 corresponds to training with the entire training set of 30, 000 images, while a fraction of 0.1 corresponds to 3, 000 images. While IWNE models deteriorate in performance, IFI models show only a marginal drop as shown in Fig 2.

The results clearly advocate the use of IFI models as opposed to not using external data, which is in line with most part of the medical imaging literature but at first sight contradicts [1], who found no improvements from IFI as compared to direct training on a considerably larger closed source DR dataset. The inferior results of IWNE-CL compared to IFI-CL can potentially be attributed to two factors: the size of Eyepacs-1 as pretraining is with around 30k samples, very small compared to large natural image datasets, such as ImageNet with 1.2M images, where self-supervised contrastive methods were demonstrated to work really well. In addition, for IWNE-CL we used the same set of transformations proposed for ImageNet in [13], which certainly represents a suboptimal choice for the DR images that differ qualitatively from natural images and the pretraining algorithm is rather sensitive to this choice.

### Statistics of eigenvalues

#### Condition number

**Fig 3:**
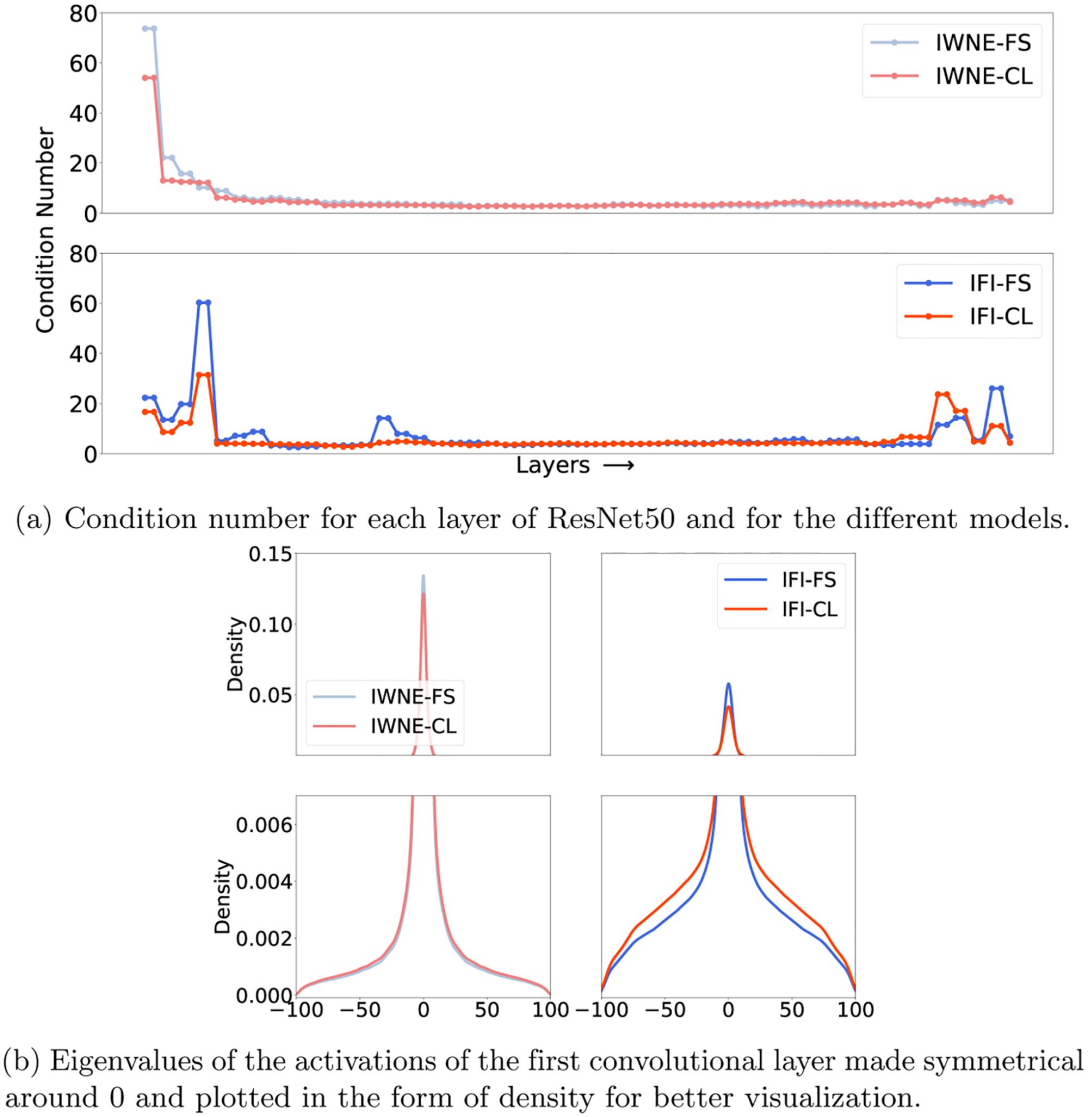
The statistics of the eigenvalues are shown here. a) shows the condition number of all the layers and b) shows the eigenvalues of the activations of the first convolutional layer. (a) Condition number for each layer of ResNet50 and for the different models. (b) Eigenvalues of the activations of the first convolutional layer made symmetrical around 0 and plotted in the form of density for better visualization.

To better understand what makes the IFI models achieve higher performance, we study the activations of the hidden layers. In particular, we compute the eigenvalues of the activations of each layer in the four models we considered. Using the eigenvalues, we plot the condition number [64] as shown in Fig 3a. To prevent the condition number from having very large values due to division by the minimum of the eigenvalues, we redefine the condition number as follows:

$$\begin{array}{c} \kappa \left(A\right)=\frac{|\lambda_{p_{99.9}}\left(A\right)|}{|\lambda_{p_{90}}\left(A\right)|} \end{array}$$ (1)

where *A* are the activations of a hidden layer, *κ*(*A*) is the condition number and $\lambda_{p_{i}}\left(A\right)$ is the eigenvalue corresponding to the *i*^*th*^ percentile of the eigenvalues. While the top row in Fig 3a shows the condition numbers of the IWNE models, the bottom row shows the condition number of the IFI models. The x-axis in both the figures corresponds to the layers of ResNet50.

We find in Fig 3a that the condition number for IFI models is much lower than that of IWNE implying significantly more diverse features learned. Also, in both versions of initializations, we find that the condition number for self-supervised learning is lower than that of supervised learning in the initial layers. This indicates that self-supervised learning extracts more diverse features than its supervised counterparts. We also find in Fig 3a that for all the different models, the condition number is flattened out and becomes indistinguishable for the latter layers. The initial layers form the crux of the learning process extracting effective and diverse feature representations while the latter layers learn to aggregate these features. On the other hand, the final layers are responsible for the discriminative classification, thus reducing the diversity here can be beneficial. We also observe this phenomenon in Fig 3a, where the conditional number of IFI models in comparison to IWNE models increase in the final layers, indicating loss in diversity that in turn leads to superior performance as reported in Fig 2.

#### Spread of eigenvalues

To investigate the distinctive aspects of the initial layers, we plot the eigenvalues of the first layer for all four models in Fig 3b. The eigenvalues are made symmetrical around 0 and plotted in the form of density to make for better visualization. The bottom row in Fig 3b also zooms in on the tails. We find that the IWNE models obtain high and peaked eigenvalues in comparison to IFI models. In addition to lower peak values, the IFI models show heavy-tailedness in comparison to that of IWNE models. Similar to the findings in the experiments on the condition number, self-supervised learning in contrast to supervised learning shows a slightly lower peak value. Additionally, in both versions of the initialization, self-supervised learning models show more heavy tailedness.

The results indicate that IWNE models learn kernels in the first convolutional layer that are activated for some very specific patterns. On the contrary, IFI models learn kernels that activate for a broader range of input features. The superior performance of IFI models can be attributed to this effect while this may also lead to several other benefits including increase in generalization and robustness.

#### Distribution fitting

In this section, we fit the eigenvalues of the first convolutional layer to the parameters of several distributions and report the distribution that fits best [65]. Among a wide range parameterized distributions, we find in Table 2 that all the four models fit best to the *Pareto* distribution, though the parameters vary. Pareto distribution with the shape parameter *α* = 1.16 corresponds to the 80−20 rule, implying that 80% of the results come from 20% of the causes [66]. IWNE models show *α* values higher than 1.16. This indicates that the overall result comes from less than 20% of the activations. In other words, the kernels learned by the IWNE models extract small number of, yet highly curated set of features from the input. In contrast, we find that IFI brings down the value of *α* for the Pareto distribution implying a wider range of feature representations learned by the first convolutional layer. Additionally, in both versions of initializations, CL shows reduced value of *α* when compared to FS indicating that the kernels learned by CL methods fire on a further broader range of input.

**Table 2:** Distribution fitting for the eigenvalues of the activations of the first layer. For all the four models, the eigenvalues are best parametrized by a Pareto distribution. We also find that the self-supervised models show smaller value for the shape parameter of the Pareto distribution.

Method	Distribution	Parameters
IWNE-FS	Pareto	*α* = 1.45
**IWNE-CL**	**Pareto**	***α* = 1.28**
IFI-FS	Pareto	*α* = 0.87
**IFI-CL**	**Pareto**	***α* = 0.73**

Our studies show that pretraining and self-supervised learning is beneficial for the downstream medical imaging task to be able learn kernels that fire broadly and in turn extract more diverse and effective features from the input.

### Robustness to distortions

The heavy-tailed activation statistics in combination with ReLU-thresholding in Section Statistics of Eigenvalues showed that a larger number of neurons are capable of detecting structures in the input when the input data is varied according to sampling from the dataset. One can expect that this also may translate to an increased detection capability when input samples are varied by data augmentation parameters towards zones of lower data density. We have performed this experiment for the IWNE and IFI models by distorting the input with a set of predefined distortions as shown in [67].

**Fig 4:**
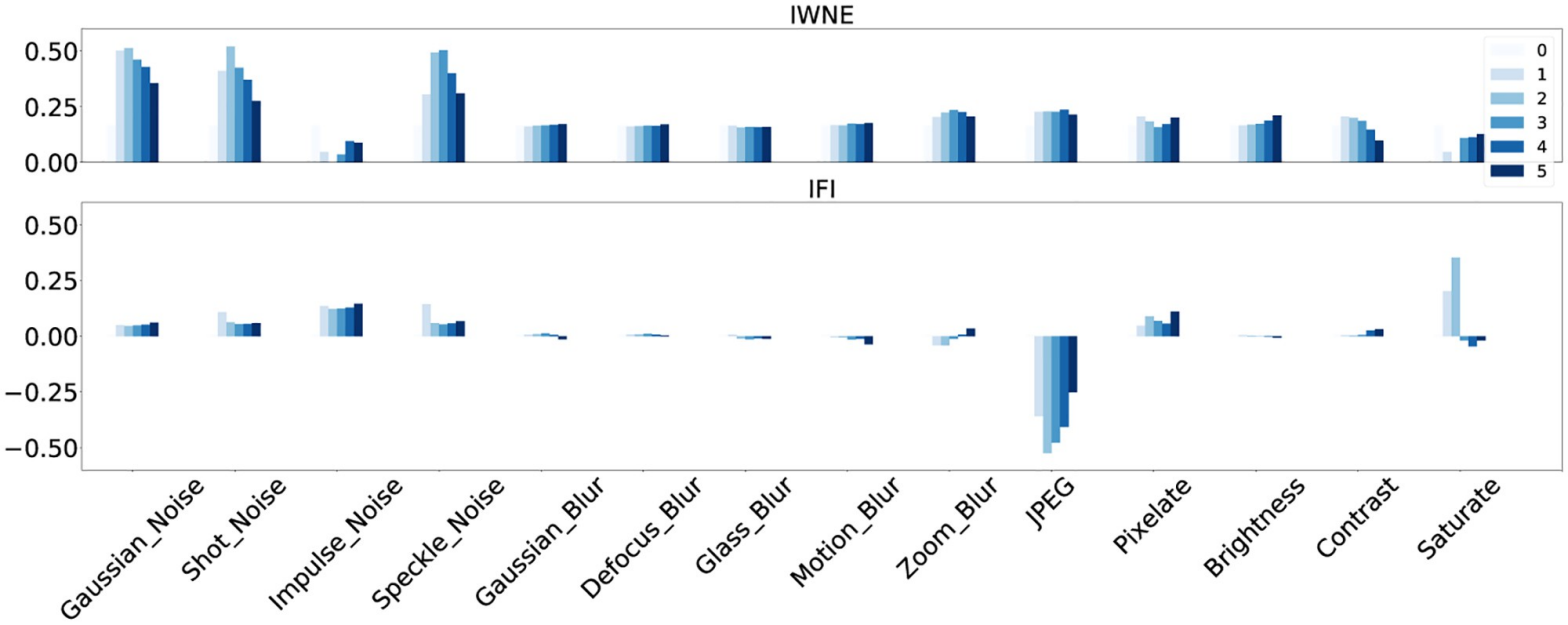
This figure shows the robustness to distortions for the different models. The difference in the softmax probabilities of the output between the CL and FS model is plotted here. The intensity of the color indicates the severity of the distortions. Top row shows the difference for IWNE models. Bottom row shows the difference for IFI models. In case of IWNE, the difference is consistently positive, implying that the self-supervised model has a higher prediction score than the plainly supervised model and thus exhibits a higher robustness to distortions. See Section Robustness to Distortions: for a detailed discussion.

One can see from Fig 4 that for the majority of distortion cases, the score for the self-supervised model is higher, indicating a higher robustness to the respective distortions. There is a marked difference between IWNE and IFI models. In the former case, CL always provides an increase in robustness in comparison to FS. Using IFI in the latter case is known to provide good generalization for finetuning with respect to a wide range of target datasets. This improved generalization levels the difference between FS and CL. However IFI-CL still improves robustness for different noise types, pixelation and lower levels of saturation changes. Note the conspicuous outlier in IFI for JPEG compression.

### Quantitative analysis of learned cues

Explainability for DNN reveals what the model looks at on the image to make the prediction [68–79]. Using ground-truth segmentation masks, explanations have been evaluated to show quantitatively if what the model is looking at, is relevant for making the decision [80]. In the case of DR, a reasonable expectation is that the trained model looks at lesions in the retina that are indicative of the disease in order to make its decision. In order to evaluate the explanation heatmaps, we use the dataset of IDRiD [11] containing detailed pixel-wise annotation of the different lesions that contribute to the disease. The dataset consists of 80 images with segmentation masks for microaneurysms, haemorrhages and hard exudates. The IDRiD dataset also contains segmentation maps for soft exudates for a smaller subset of images, which we excluded from our quantitative evaluation.

To obtain explanation heatmaps, we utilize Layer-wise Relevance Propagation (LRP) [70, 74]. LRP is a principled approach to decompose the decisions of the classifier and assign pixel-wise relevances determining the contributions of the input pixels towards the decision. The layer-wise conservation principle in LRP assures that the relevances from a higher layer is preserved when propagated to a lower layer $\sum_{i}R_{i}^{\left(l\right)}=\sum_{j}R_{j}^{\left(l+1\right)}$. The forward pass for the activations of any given layer in a DNN can be defined as be the weighted activation of neuron *i* onto neuron *j* in the next layer. Let $z_{ij}=a_{i}^{l}w_{ij}^{\left(l,l+1\right)}$, where $a_{i}^{l}$ is the activation of a neuron *i* in the previous layer, and where *z*_*ij*_ is the contribution of neuron *i* at layer *l* to the activation of the neuron *j* at layer *l* + 1. The relevances are computed using the *α*_1_ *β*_0_ rule:

$$\begin{array}{c} R_{i}=\sum_{j}(\alpha \cdot \frac{z_{ij}^{+}}{\sum_{i^{\prime }}z_{i^{\prime }j}^{+}}+\beta \cdot \frac{z_{ij}^{-}}{\sum_{i^{\prime }}z_{i^{\prime }j}^{-}})R_{j} \end{array}$$ (2)

The intuition behind LRP is that neurons of the lower layers that mostly contribute to the activations of the higher layer neuron receive a larger share of the relevance *R*_*j*_ of the neuron *j*. Decomposing the relevances into its positive part $z_{ij}^{+}$ and the negative part $z_{ij}^{-}$ allows for exact conservation of the relevances [69].

**Fig 5:**
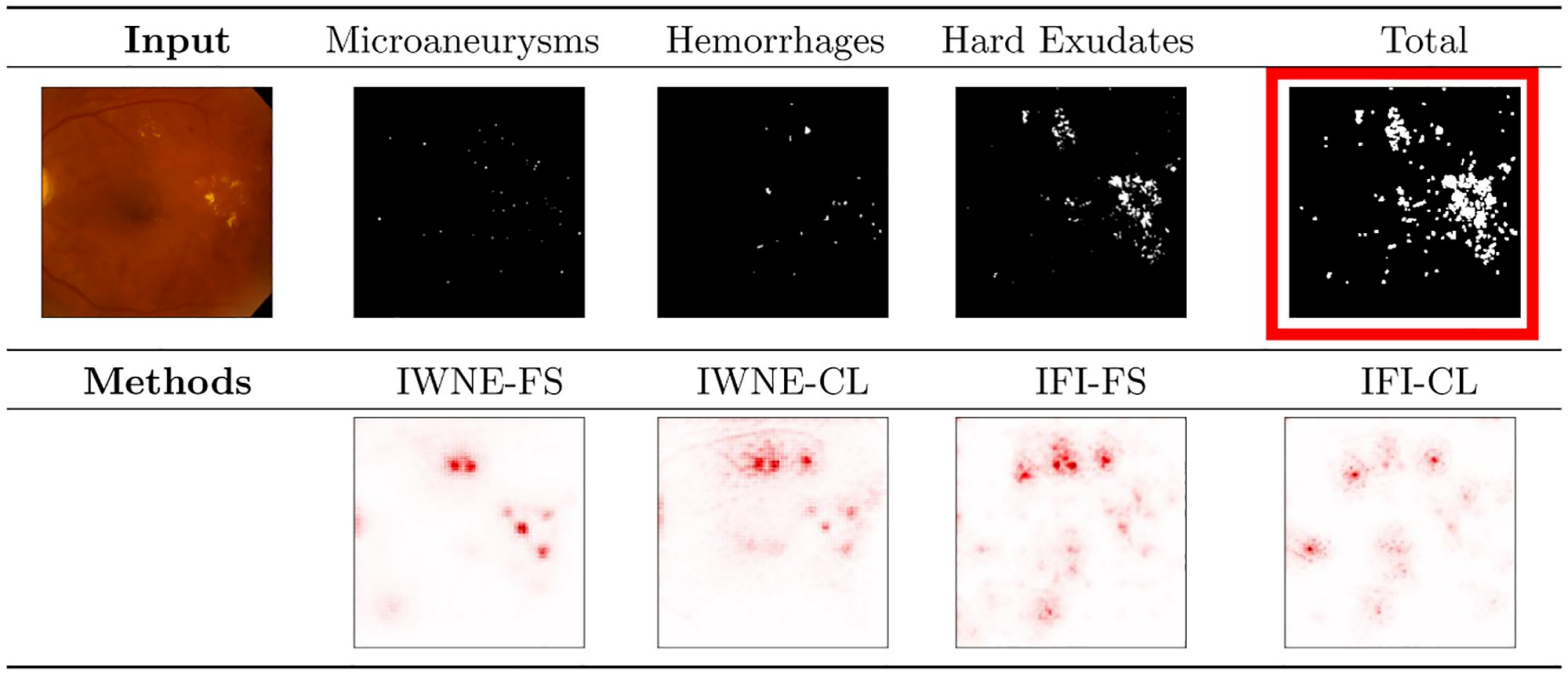
Top left image in the figure shows the input followed by the segmentation maps from the IDRiD dataset. Top right image is the total that we compute by combining the segmentation maps of different lesions. Bottom row shows the explanation heatmaps for the given input. Each explanation heatmap is correlated with the total image marked in red to evaluate the effectiveness of the model towards making the prediction for the disease. We find that IWNE-FS overfits on the hard exudates and also fails to pick up on cues related to microaneurysms. We also find that explanation heatmaps of IFI models show reduced signs of overfitting to a single lesion when compared to IWNE.

Fig 5 shows the input followed by the segmentation maps for different lesions in the top row. The final image in the top row combines the different lesions to form the total. The bottom row shows the explanation heatmaps by using the different training methods. By comparing each result to the total marked in red in Fig 5, we can evaluate the effectiveness of the model in looking at the lesion to make the prediction. We find that explanation heatmaps from IWNE overfit on the hard exudates and show minimal correlation with the other lesions. On the other hand, explanation heatmaps from IFI models are significantly more outspread correlating better with different lesions.

The correlation of explanation heatmaps to the ground-truth segmentation maps also helps us make a quantitative evaluation of how accurately the models relies on the disease to make its prediction. We follow the evaluating strategies adopted in [80] including relevance mass accuracy and relevance rank accuracy. For a given input RGB image *x*, relevances *R*_*i*_ determining the importance of the input features *x*_*i*_ are also in the dimensions of the image. However, the the ground truth segmentation mask *S* ⊆ [0, 1] are only in two dimensions. Hence, we pool the relevances across the channels to be able to compare them with the segmentation masks. We utilize the two pooling strategies followed by [80]:

- $\text{sum\_pos}:\;R^{pool}=max\left(0,\sum_{k=1}^{C}R_{k}\right)$
- $\text{l2\_norm\_sq}:\;R^{pool}=\sum_{k=1}^{C}{R_{k}}^{2}$

where *C* is the number of channels. However, the findings here are agnostic to the pooling strategy utilized. Given pooled relevances and ground truth segmentation masks, the relevance mass accuracy is defined as:

$$\begin{array}{c} \text{RMA}=\frac{\sum_{i\in S}R_{i}^{pool}}{\sum_{i}R_{i}^{pool}} \end{array}$$ (3)

where the numerator corresponds to the sum of relevances where the ground truth segmentation maps exists and the denominator is the sum of all relevances. The relevance rank accuracy is defined as:

$$\begin{array}{c} \text{RRA}=\frac{|R_{p_{i}}^{pool}\cap S|}{\left|S\right|} \end{array}$$ (4)

where $R_{p_{i}}^{pool}$ is the relevances in the top *i*^*th*^ percentile. While RMA corresponds to the precision, RRA corresponds to the recall.

Table 3 shows the results for RMA and RRA for the explanation heatmaps correlated with the ground-truth segmentation maps from the IDRiD challenge. We report the accuracies for each lesion—microaneurysms, haemorrhages and hard exudates and a total, where we combine the above mentioned lesions. The heatmaps for each of the methods are computed by backpropagating from the output neuron corresponding to severe DR, which can also be considered as the ground truth DR level for the given input. The heatmaps are evaluated using the two pooling strategies mentioned above for each lesion. As a control, we also report the results by replacing explanation heatmaps with random variables from Gaussian distribution. Any method that shows similar results to the control indicates that the heatmaps are just random, i.e. the model looks at random set of input features to make its prediction. In each category (lesion), the best result among the different training strategies are marked in bold for each pooling method.

**Table 3:** Relevance mass accuracy (RMA) and relevance rank accuracy (RRA) on the LRP-*α*_1_*β*_0_ explanation heatmaps of images of the IDRiD dataset. The results show that while supervised models overfit on the hard exudates, the self-supervised models look at diverse set of input features (lesions). On the other hand, we also find that IFI models show higher accuracies when compared to IWNE models.

Lesions	Method	Pooling	RMA				RRA			
			Random		LRP-*α*_1_*β*_0_		Random		LRP-*α*_1_*β*_0_	
			Mean	Median	Mean	Median	Mean	Median	Mean	Median
Microaneurysms	IWNE-FS	sum_pos	0.0073	0.0064	0.0076	0.0072	0.9885	0.9895	0.3447	0.4146
		l2_norm_sq	0.0074	0.0067	0.0041	0.0025	0.9894	0.9900	0.4888	0.5047
	IWNE-CL	sum_pos	0.0074	0.0064	**0.0093**	**0.0077**	0.9879	0.9901	**0.4370**	**0.5224**
		l2_norm_sq	0.0073	0.0064	**0.0075**	**0.0042**	0.9882	0.9912	**0.5777**	**0.5736**
	IFI-FS	sum_pos	0.0073	0.0061	0.0172	0.0143	0.9913	0.9922	0.5097	0.5551
		l2_norm_sq	0.0074	0.0068	0.0374	0.0218	0.9891	0.9900	0.5705	0.5713
	IFI-CL	sum_pos	0.0073	0.0061	**0.0198**	**0.0186**	0.9896	0.9897	**0.5831**	**0.6251**
		l2_norm_sq	0.0073	0.0067	**0.0595**	**0.0381**	0.9902	0.9917	**0.6357**	**0.6366**
Haemorrhages	IWNE-FS	sum_pos	0.0234	0.0130	0.0251	0.0165	0.9902	0.9911	0.3845	0.4547
		l2_norm_sq	0.0233	0.0126	0.0139	0.0056	0.9880	0.9905	0.5414	0.5565
	IWNE-CL	sum_pos	0.0232	0.0126	**0.0602**	**0.0458**	0.9889	0.9904	**0.4971**	**0.6076**
		l2_norm_sq	0.0234	0.0125	**0.1063**	**0.0525**	0.9881	0.9892	**0.6357**	**0.6371**
	IFI-FS	sum_pos	0.0233	0.0126	0.0711	0.0578	0.9896	0.9895	0.5840	0.6127
		l2_norm sq	0.0233	0.0125	0.1438	0.1243	0.9891	0.9904	0.6571	0.6551
	IFI-CL	sum_pos	0.0234	0.0127	**0.0765**	**0.0722**	0.9897	0.9911	**0.6898**	**0.7194**
		l2_norm_sq	0.0234	0.0125	**0.1874**	**0.1808**	0.9873	0.9911	**0.7403**	**0.7405**
Hard Exudates	IWNE-FS	sum_pos	0.0409	0.0195	**0.1954**	**0.1734**	0.9897	0.9906	0.5086	**0.6959**
		l2_norm sq	0.0409	0.0190	**0.4201**	**0.4921**	0.9898	0.9903	**0.7114**	**0.7435**
	IWNE-CL	sum_pos	0.0409	0.0200	0.1206	0.1018	0.9889	0.9898	**0.5136**	0.5887
		l2_norm_sq	0.0409	0.0191	0.2338	0.1652	0.9892	0.9898	0.6656	0.7038
	IFI-FS	sum_pos	0.0408	0.0195	**0.1103**	**0.0861**	0.9895	0.9905	**0.5480**	**0.6258**
		l2_norm_sq	0.0410	0.0194	**0.2125**	**0.1659**	0.9905	0.9915	**0.6125**	**0.6561**
	IFI-CL	sum_pos	0.0409	0.0188	0.0725	0.0533	0.9890	0.9896	0.5425	0.5762
		l2_norm_sq	0.0412	0.0193	0.1195	0.0858	0.9888	0.9903	0.6088	0.6260
Total	IWNE-FS	sum_pos	0.0710	0.0558	**0.2266**	**0.2000**	0.9899	0.9905	0.4459	0.5655
		l2_norm_sq	0.0711	0.0563	**0.4363**	**0.5104**	0.9893	0.9898	0.6151	**0.6503**
	IWNE-CL	sum_pos	0.0710	0.0565	0.1887	0.1619	0.9884	0.9891	**0.5015**	**0.6083**
		l2_norm_sq	0.0711	0.0565	0.3457	0.3330	0.9884	0.9890	**0.6459**	0.6334
	IFI-FS	sum_pos	0.0709	0.0561	**0.1969**	**0.1886**	0.9893	0.9897	0.5479	0.5941
		l2_norm_sq	0.0711	0.0557	**0.3905**	**0.3964**	0.9893	0.9896	0.6150	0.6245
	IFI-CL	sum_pos	0.0711	0.0576	0.1671	0.1724	0.9893	0.9896	**0.5847**	**0.6144**
		l2_norm_sq	0.0713	0.0569	0.3625	0.3650	0.9895	0.9897	**0.6428**	**0.6463**

We find in Table 3 that in the case of microaneurysms, random explanations achieve a mean accuracy of 0.0073 for RMA. Here, the model IWNE-FS achieves results that is very close to the results for the random explanations. On the other hand, all the other models report accuracies that are higher than the corresponding control value. This indicates that IWNE-FS may be ignoring microaneurysms for making its decision. The RMA results in Table 3 show that for the IWNE models, CL achieves better results. IFI models, in general report higher accuracies than that of IWNE models. Similar to IWNE, we find for IFI models that CL reports better RMA than FS using both the pooling strategies. This is confirmed again with results of RRA in the same table, where models with CL achieves the best results. Microaneurysms are the smallest lesions and it is vital for a method to base its decision on them for detecting progressive cases of DR. Our results indicate that IFI models and CL in particular are better equipped at including microaneurysms to make their predictions.

Haemorrhages are lesions that are slightly larger than microaneurysms. We find in Table 3 that here again IWNE-FS reports similar accuracies to that of the control indicating that this model may be ignoring the haemorrhages as well. Among IWNE models, CL clearly achieves higher RMA as well as higher RRA. This is again the case on the IFI models where CL achieves higher RMA and RRA indicating that the explanations using this model are better correlated with the ground-truth than their supervised counterpart FS.

In contrast to the smaller lesions, the hard exudates are large yellowish white deposits with sharp gradients. Here for RMA, the supervised models achieve better results than the self-supervised models as shown in Table 3. The results on RRA for hard exudates show that on majority of the cases, for both IWNE and IFI models, the supervised models show higher accuracies than the self-supervised models.

For the *total*, which measures the sum of the all the different lesions, we find here again that the supervised models achieve better results with RMA as shown in 3. With RRA, the IWNE models do not clearly outperform each other in the case of total. However, for IFI, the self-supervised model clearly outperforms the supervised model for the total of all the lesions.

The results of RMA and RRA in Table 3 reveal that the supervised models overfit on the hard exudates in both versions of initializations. IWNE-FS in particular fails to base its decision on microaneurysms and haemorrhages that may be highly relevant for the prediction of onset of the disease. The results on the total are skewed by the results on the hard exudates. In alignment with our observations in Section Statistics of Eigenvalues, we find that the IFI models look at diverse set of input features (lesions) and report consistently higher accuracies than their IWNE counterparts. Among IFI, the results of CL correlates better with the explanation heatmaps for a variety of lesions indicating that they look at more diverse set of input features than any other method.

## Summary and conclusions

Deep learning-based methods for the diagnosis of diabetic retinopathy have shown remarkable performance. In our paper, we study the important question of the robustness of different training strategies—namely initialization from ImageNet pretraining and self-supervised learning. Our findings are three-fold: Firstly, we show the performance gains obtained by self-supervised learning in diabetic retinopathy. Secondly, we demonstrate the advantage of self-supervised learning along with initialization from ImageNet pretraining for diabetic retinopathy by analyzing the statistics of the eigenvalues of the feature representations learned. We also show improvements in robustness to distortions for self-supervised learning in comparison to purely supervised training. Finally, we use interpretability methods to gain quantitative insights into the patterns exploited by models trained using the different training schemes. In particular, we find that initialization from ImageNet pretraining significantly reduces overfitting to large lesions along with improvements in taking into account minute lesions, which are indicative of the progression of the disease.

With our study, we try to convey that a more holistic view on the benefits of pretraining and self-supervision in medical imaging along the lines of the present study is important. To summarize, in absence of large unlabeled domain-specific data that would allow for self-supervised pretraining, we see numerous benefits in favor of using self-supervised pretrained models on ImageNet as starting point for finetuning on domain-specific data, which we put as a general recommendation.

## References

[1] Raghu M, Zhang C, Kleinberg J, Bengio S. Transfusion: Understanding transfer learning for medical imaging. In: Advances in neural information processing systems; 2019. p. 3347–3357.

[2] Gulshan V, Peng L, Coram M, Stumpe MC, Wu D, Narayanaswamy A, et al. Development and validation of a deep learning algorithm for detection of diabetic retinopathy in retinal fundus photographs. Jama. 2016;316(22):2402–2410. doi: 10.1001/jama.2016.17216 27898976

[3] Voets M, Møllersen K, Bongo LA. Reproduction study using public data of: Development and validation of a deep learning algorithm for detection of diabetic retinopathy in retinal fundus photographs. PLOS ONE. 2019;14(6):e0217541. doi: 10.1371/journal.pone.0217541 31170223 PMC6553744

[4] Sowrirajan H, Yang J, Ng AY, Rajpurkar P. MoCo pretraining improves representation and transferability of chest X-ray models. arXiv preprint arXiv:201005352. 2020;.

[5] Chen T, Kornblith S, Swersky K, Norouzi M, Hinton GE. Big Self-Supervised Models are Strong Semi-Supervised Learners. In: Advances in Neural Information Processing Systems. vol. 33; 2020. p. 22243–22255.

[6] Azizi S, Mustafa B, Ryan F, Beaver Z, Freyberg J, Deaton J, et al. Big Self-Supervised Models Advance Medical Image Classification. arXiv preprint arXiv:210105224. 2021;.

[7] Binder A, Bockmayr M, Hägele M, Wienert S, Heim D, Hellweg K, et al. Morphological and molecular breast cancer profiling through explainable machine learning. Nature Machine Intelligence. 2021;3(4):355–366. doi: 10.1038/s42256-021-00303-4

[8] Neyshabur B, Sedghi H, Zhang C. What is being transferred in transfer learning? In: Advances in Neural Information Processing; 2020.

[9] Deng J, Dong W, Socher R, Li LJ, Li K, Fei-Fei L. Imagenet: A large-scale hierarchical image database. In: Computer Vision and Pattern Recognition; 2009. p. 248–255.

[10] Kaggle. Diabetic Retinopathy Detection Challenge;. https://www.kaggle.com/c/diabetic-retinopathy-detection.

[11] Porwal P, Pachade S, Kokare M, Deshmukh G, Son J, Bae W, et al. IDRiD: Diabetic Retinopathy–Segmentation and Grading Challenge. Medical Image Analysis. 2020;59:101561. doi: 10.1016/j.media.2019.101561 31671320

[12] He K, Fan H, Wu Y, Xie S, Girshick R. Momentum contrast for unsupervised visual representation learning. In: Computer Vision and Pattern Recognition; 2020. p. 9729–9738.

[13] Chen T, Kornblith S, Norouzi M, Hinton G. A simple framework for contrastive learning of visual representations. In: International Conference on Machine Learning; 2020. p. 1597–1607.

[14] Grill JB, Strub F, Altché F, Tallec C, Richemond PH, Buchatskaya E, et al. Bootstrap your own latent: A new approach to self-supervised Learning. In: Advances in Neural Information Processing Systems; 2020.

[15] Caron M, Misra I, Mairal J, Goyal P, Bojanowski P, Joulin A. Unsupervised Learning of Visual Features by Contrasting Cluster Assignments. In: Advances in Neural Information Processing Systems; 2020.

[16] Sowrirajan H, Yang J, Ng AY, Rajpurkar P. MoCo pretraining improves representation and transferability of chest X-ray models. In: Medical Imaging with Deep Learning; 2021.

[17] Sriram A, Muckley M, Sinha K, Shamout F, Pineau J, Geras KJ, et al. COVID-19 Deterioration Prediction via Self-Supervised Representation Learning and Multi-Image Prediction. arXiv preprint arXiv:210104909. 2021;. 33469559

[18] Irvin J, Rajpurkar P, Ko M, Yu Y, Ciurea-Ilcus S, Chute C, et al. Chexpert: A large chest radiograph dataset with uncertainty labels and expert comparison. In: AAAI Conference on Artificial Intelligence. vol. 33; 2019. p. 590–597.

[19] Liu Y, Jain A, Eng C, Way DH, Lee K, Bui P, et al. A deep learning system for differential diagnosis of skin diseases. Nature Medicine. 2020;26(6):900–908. doi: 10.1038/s41591-020-0842-3 32424212

[20] Takahashi H, Tampo H, Arai Y, Inoue Y, Kawashima H. Applying artificial intelligence to disease staging: Deep learning for improved staging of diabetic retinopathy. PLOS ONE. 2017;12(6):e0179790. doi: 10.1371/journal.pone.0179790 28640840 PMC5480986

[21] Gargeya R, Leng T. Automated identification of diabetic retinopathy using deep learning. Ophthalmology. 2017;124(7):962–969. doi: 10.1016/j.ophtha.2017.02.008 28359545

[22] Lam C, Yu C, Huang L, Rubin D. Retinal lesion detection with deep learning using image patches. Investigative ophthalmology & visual science. 2018;59(1):590–596. doi: 10.1167/iovs.17-22721 29372258 PMC5788045

[23] Lam C, Yi D, Guo M, Lindsey T. Automated detection of diabetic retinopathy using deep learning. AMIA summits on translational science proceedings. 2018;2018:147. 29888061 PMC5961805

[24] Gao Z, Li J, Guo J, Chen Y, Yi Z, Zhong J. Diagnosis of diabetic retinopathy using deep neural networks. IEEE Access. 2018;7:3360–3370.

[25] Wang X, Lu Y, Wang Y, Chen WB. Diabetic retinopathy stage classification using convolutional neural networks. In: International Conference on Information Reuse and Integration; 2018. p. 465–471.

[26] Wan S, Liang Y, Zhang Y. Deep convolutional neural networks for diabetic retinopathy detection by image classification. Computers & Electrical Engineering. 2018;72:274–282. doi: 10.1016/j.compeleceng.2018.07.042

[27] Chen H, Zeng X, Luo Y, Ye W. Detection of Diabetic Retinopathy using Deep Neural Network. In: International Conference on Digital Signal Processing; 2018. p. 1–5.

[28] Johari MH, Hassan HA, Yassin AIM, Tahir NM, Zabidi A, Rizman ZI, et al. Early detection of diabetic retinopathy by using deep learning neural network. International Journal of Engineering and Technology. 2018;7(4):198–201. doi: 10.14419/ijet.v7i4.11.20804

[29] Xu X, Lin J, Tao Y, Wang X. An Improved DenseNet Method Based on Transfer Learning for Fundus Medical Images. In: International Conference on Digital Home; 2018. p. 137–140.

[30] Poplin R, Varadarajan AV, Blumer K, Liu Y, McConnell MV, Corrado GS, et al. Prediction of cardiovascular risk factors from retinal fundus photographs via deep learning. Nature Biomedical Engineering. 2018;2(3):158–164. doi: 10.1038/s41551-018-0195-0 31015713

[31] Varadarajan AV, Poplin R, Blumer K, Angermueller C, Ledsam J, Chopra R, et al. Deep learning for predicting refractive error from retinal fundus images. Investigative Ophthalmology & Visual Science. 2018;59(7):2861–2868. doi: 10.1167/iovs.18-23887 30025129

[32] Rakhlin A. Diabetic Retinopathy detection through integration of Deep Learning classification framework. bioRxiv. 2018; p. 225508.

[33] Zhang W, Zhong J, Yang S, Gao Z, Hu J, Chen Y, et al. Automated identification and grading system of diabetic retinopathy using deep neural networks. Knowledge-Based Systems. 2019;175:12–25. doi: 10.1016/j.knosys.2019.03.016

[34] Zeng X, Chen H, Luo Y, Ye W. Automated diabetic retinopathy detection based on binocular siamese-like convolutional neural network. IEEE Access. 2019;7:30744–30753. doi: 10.1109/ACCESS.2019.2903171

[35] Bajwa MN, Taniguchi Y, Malik MI, Neumeier W, Dengel A, Ahmed S. Combining Fine-and Coarse-Grained Classifiers for Diabetic Retinopathy Detection. In: Medical Image Understanding and Analysis; 2019. p. 242–253.

[36] Pires R, Avila S, Wainer J, Valle E, Abramoff MD, Rocha A. A data-driven approach to referable diabetic retinopathy detection. Artificial Intelligence in Medicine. 2019;96:93–106. doi: 10.1016/j.artmed.2019.03.009 31164214

[37] Grzybowski A, Brona P, Lim G, Ruamviboonsuk P, Tan GS, Abramoff M, et al. Artificial intelligence for diabetic retinopathy screening: a review. Eye. 2020;34(3):451–460. doi: 10.1038/s41433-019-0566-0 31488886 PMC7055592

[38] Samanta A, Saha A, Satapathy SC, Fernandes SL, Zhang YD. Automated detection of diabetic retinopathy using convolutional neural networks on a small dataset. Pattern Recognition Letters. 2020;135:293–298. doi: 10.1016/j.patrec.2020.04.026

[39] Wang J, Bai Y, Xia B. Simultaneous Diagnosis of Severity and Features of Diabetic Retinopathy in Fundus Photography Using Deep Learning. IEEE Journal of Biomedical and Health Informatics. 2020;24(12):3397–3407. doi: 10.1109/JBHI.2020.3012547 32750975

[40] Ludwig CA, Perera C, Myung D, Greven MA, Smith SJ, Chang RT, et al. Automatic identification of referral-warranted diabetic retinopathy using deep learning on mobile phone images. Translational Vision Science & Technology. 2020;9(2):60–60. doi: 10.1167/tvst.9.2.60 33294301 PMC7718806

[41] Alyoubi WL, Shalash WM, Abulkhair MF. Diabetic retinopathy detection through deep learning techniques: A review. Informatics in Medicine Unlocked. 2020;20:100377. doi: 10.1016/j.imu.2020.100377

[42] Ruamviboonsuk P, Tiwari R, Sayres R, Nganthavee V, Hemarat K, Kongprayoon A, et al. Real-time diabetic retinopathy screening by deep learning in a multisite national screening programme: a prospective interventional cohort study. The Lancet Digital Health. 2022;4(4):e235–e244. doi: 10.1016/S2589-7500(22)00017-6 35272972

[43] Leibig C, Allken V, Ayhan MS, Berens P, Wahl S. Leveraging uncertainty information from deep neural networks for disease detection. Scientific Reports. 2017;7(1). doi: 10.1038/s41598-017-17876-z 29259224 PMC5736701

[44] Filos A, Farquhar S, Gomez AN, Rudner TG, Kenton Z, Smith L, et al. A systematic comparison of bayesian deep learning robustness in diabetic retinopathy tasks. arXiv preprint arXiv:191210481. 2019;.

[45] Sayres R, Taly A, Rahimy E, Blumer K, Coz D, Hammel N, et al. Using a Deep Learning Algorithm and Integrated Gradients Explanation to Assist Grading for Diabetic Retinopathy. Ophthalmology. 2019;126(4):552–564. doi: 10.1016/j.ophtha.2018.11.016 30553900

[46] Messidor 2;. http://www.adcis.net/en/third-party/messidor2/.

[47] Taleb A, Loetzsch W, Danz N, Severin J, Gaertner T, Bergner B, et al. 3D Self-Supervised Methods for Medical Imaging. In: Advances in Neural Information Processing; 2020.

[48] Holmberg OG, Köhler ND, Martins T, Siedlecki J, Herold T, Keidel L, et al. Self-supervised retinal thickness prediction enables deep learning from unlabelled data to boost classification of diabetic retinopathy. Nature Machine Intelligence. 2020;2(11):719–726. doi: 10.1038/s42256-020-00247-1

[49] Geirhos R, Narayanappa K, Mitzkus B, Bethge M, Wichmann FA, Brendel W. On the surprising similarities between supervised and self-supervised models. arXiv preprint arXiv:201008377. 2020;.

[50] Navarro F, Watanabe C, Shit S, Sekuboyina A, Peeken JC, Combs SE, et al.. Evaluating the Robustness of Self-Supervised Learning in Medical Imaging; 2021.

[51] Hendrycks D, Lee K, Mazeika M. Using Pre-Training Can Improve Model Robustness and Uncertainty. In: International Conference on Machine Learning; 2019. p. 2712–2721.

[52] Hendrycks D, Mazeika M, Kadavath S, Song D. Using self-supervised learning can improve model robustness and uncertainty. In: Advances in Neural Information Processing Systems. vol. 32; 2019. p. 15663–15674.

[53] Hendrycks D, Liu X, Wallace E, Dziedzic A, Krishnan R, Song D. Pretrained Transformers Improve Out-of-Distribution Robustness. In: Association for Computational Linguistics; 2020. p. 2744–2751. doi: 10.18653/v1/2020.acl-main.244

[54] Djolonga J, Yung J, Tschannen M, Romijnders R, Beyer L, Kolesnikov A, et al. On robustness and transferability of convolutional neural networks. arXiv preprint arXiv:200708558. 2020;.

[55] Jiang Z, Chen T, Chen T, Wang Z. Robust Pre-Training by Adversarial Contrastive Learning. In: Advances in Neural Information Processing. vol. 33; 2020. p. 16199–16210.

[56] Chen T, Liu S, Chang S, Cheng Y, Amini L, Wang Z. Adversarial Robustness: From Self-Supervised Pre-Training to Fine-Tuning. In: Computer Vision and Pattern Recognition; 2020. p. 699–708.

[57] Peng AY, Koh YS, Riddle P, Pfahringer B. Using supervised pretraining to improve generalization of neural networks on binary classification problems. In: Joint European Conference on Machine Learning and Knowledge Discovery in Databases; 2018. p. 410–425.

[58] Chen S, Ma K, Zheng Y. Med3d: Transfer learning for 3d medical image analysis. arXiv preprint arXiv:190400625. 2019;.

[59] Chen T, Frankle J, Chang S, Liu S, Zhang Y, Carbin M, et al. The Lottery Tickets Hypothesis for Supervised and Self-supervised Pre-training in Computer Vision Models. arXiv preprint arXiv:201206908. 2020;.

[60] Kandel I, Castelli M. Transfer Learning with Convolutional Neural Networks for Diabetic Retinopathy Image Classification. A Review. Applied Sciences. 2020;10(6):2021. doi: 10.3390/app10062021

[61] He K, Zhang X, Ren S, Sun J. Deep residual learning for image recognition. In: Proceedings of the IEEE conference on computer vision and pattern recognition; 2016. p. 770–778.

[62] Chen X, Fan H, Girshick R, He K. Improved baselines with momentum contrastive learning. arXiv preprint arXiv:200304297. 2020;.

[63] Loshchilov I, Hutter F. Decoupled Weight Decay Regularization. In: International Conference on Learning Representations; 2019.

[64] Goodfellow I, Bengio Y, Courville A. Deep Learning. MIT Press; 2016.

[65] Taskesen E. distfit; 2019. https://github.com/erdogant/distfit.

[66] Pareto V. Cours d’économie politique. vol. 1. Librairie Droz; 1964.

[67] Hendrycks D, Dietterich T. Benchmarking Neural Network Robustness to Common Corruptions and Perturbations. Proceedings of the International Conference on Learning Representations. 2019;.

[68] Bach S, Binder A, Montavon G, Klauschen F, Müller KR, Samek W. On Pixel-wise Explanations for Non-Linear Classifier Decisions by Layer-wise Relevance Propagation. PLOS ONE. 2015;10(7):e0130140. doi: 10.1371/journal.pone.0130140 26161953 PMC4498753

[69] Samek W, Binder A, Montavon G, Lapuschkin S, Müller KR. Evaluating the visualization of what a Deep Neural Network has learned. IEEE Transactions on Neural Networks and Learning Systems. 2017;28(11):2660–2673. doi: 10.1109/TNNLS.2016.2599820 27576267

[70] Montavon G, Samek W, Müller KR. Methods for Interpreting and Understanding Deep Neural Networks. Digital Signal Processing. 2018;73:1–15. doi: 10.1016/j.dsp.2017.10.011

[71] Samek W, Wiegand T, Müller KR. Explainable Artificial Intelligence: Understanding, Visualizing and Interpreting Deep Learning Models. ITU Journal: ICT Discoveries—Special Issue 1—The Impact of Artificial Intelligence (AI) on Communication Networks and Services. 2018;1(1):39–48.

[72] Sundararajan M, Taly A, Yan Q. Axiomatic attribution for deep networks. In: International Conference on Machine Learning; 2017. p. 3319–3328.

[73] Shrikumar A, Greenside P, Kundaje A. Learning important features through propagating activation differences. In: International Conference on Machine Learning; 2017. p. 3145–3153.

[74] Samek W, Montavon G, Lapuschkin S, Anders CJ, Müller KR. Explaining Deep Neural Networks and Beyond: A Review of Methods and Applications. Proceedings of the IEEE. 2021;109(3):247–278. doi: 10.1109/JPROC.2021.3060483

[75] Samek W, Montavon G, Vedaldi A, Hansen LK, Müller KR, editors. Explainable AI: Interpreting, Explaining and Visualizing Deep Learning. vol. 11700; 2019.

[76] Hägele M, Seegerer P, Lapuschkin S, Bockmayr M, Samek W, Klauschen F, et al. Resolving challenges in deep learning-based analyses of histopathological images using explanation methods. Scientific reports. 2020;10(1):6423. doi: 10.1038/s41598-020-62724-2 32286358 PMC7156509

[77] Holzinger A, Langs G, Denk H, Zatloukal K, Müller H. Causability and explainability of artificial intelligence in medicine. Wiley Interdisciplinary Reviews: Data Mining and Knowledge Discovery. 2019;9(4):e1312. doi: 10.1002/widm.1312 32089788 PMC7017860

[78] Holzinger A, Goebel R, Mengel M, Müller H. Artificial Intelligence and Machine Learning for Digital Pathology: State-of-the-art and Future Challenges. vol. 12090. Springer Nature; 2020.

[79] Holzinger A, Malle B, Saranti A, Pfeifer B. Towards multi-modal causability with Graph Neural Networks enabling information fusion for explainable AI. Information Fusion. 2021;71:28–37. doi: 10.1016/j.inffus.2021.01.008

[80] Arras L, Osman A, Samek W. CLEVR-XAI: A Benchmark Dataset for the Ground Truth Evaluation of Neural Network Explanations. Information Fusion. 2022;81:14–40. doi: 10.1016/j.inffus.2021.11.008

[81] Paszke A, Gross S, Massa F, Lerer A, Bradbury J, Chanan G, et al. PyTorch: An Imperative Style, High-Performance Deep Learning Library. In: Advances in Neural Information Processing Systems; 2019. p. 8024–8035.

